# Endometrial Stromal Sarcoma–Associated Hypereosinophilia: A Case Report

**DOI:** 10.1155/crom/5586309

**Published:** 2025-07-09

**Authors:** Smriti Nair, Sanjna Shelukar, Sydney Kornbleuth, Grant Gillan, Emily Hansinger, Ruben Rhoades, Lakshmi Ravindran, Timothy Kuchera

**Affiliations:** ^1^Department of Medicine, Sidney Kimmel Medical College of Thomas Jefferson University, Philadelphia, Pennsylvania, USA; ^2^Department of Pathology, Sidney Kimmel Medical College of Thomas Jefferson University, Philadelphia, Pennsylvania, USA; ^3^Division of Hematology, Department of Medicine, Thomas Jefferson University, Philadelphia, Pennsylvania, USA; ^4^Division of Hospital Medicine, Department of Medicine, Sidney Kimmel Medical College of Thomas Jefferson University, Philadelphia, Pennsylvania, USA

**Keywords:** case report, endometrial stromal sarcoma, eosinophilia, HGESS

## Abstract

Eosinophilia is a common systemic reaction to allergy, parasitic infection, or drug hypersensitivity. Rarely, it manifests as a paraneoplastic phenomenon, most commonly secondary to hematologic malignancies or extensive metastatic disease in solid tumors. There is scarce literature attributing peripheral eosinophilia to solid organ malignancies, especially gynecologic malignancies. We present the first reported case of peripheral eosinophilia secondary to high-grade endometrial stromal sarcoma (HGESS). A postmenopausal woman presented with weakness, urinary incontinence, and marked peripheral eosinophilia. An unremarkable infectious workup prompted further imaging, which revealed a uterine mass. She underwent total hysterectomy with bilateral salpingo-oophorectomy, after which her eosinophilia resolved. Histopathology confirmed HGESS. One month later, the patient re-presented with recurrent eosinophilia and was found to have new metastatic lesions on CT abdomen/pelvis. She elected to pursue hospice care. This case highlights a rare and atypical presentation of an aggressive uterine malignancy underscoring peripheral eosinophilia as a potential marker of underlying malignancy.

## 1. Introduction

Uterine sarcoma is a rare form of mesenchymal malignancy, making up 2%–5% of patients with uterine malignancy. Endometrial stromal sarcomas (ESSs) are rare malignant tumors that comprise only 0.2% of all uterine malignancies originating from endometrial stromal cells [1]. While arising from the endometrium, the bulk of the tumor mass is typically found within the myometrium. Typically, ESS has the clinical presentation of pelvic pain, dysmenorrhea, and abnormal uterine bleeding, although around 25% of patients can be asymptomatic [2]. Symptoms of ESS can also be related to external compression from mass effect and metastases. As such, the patient described in this case had endorsed urinary incontinence that had worsened just before her presentation. MRI can be useful for a preoperative diagnosis, looking for bands of low signal intensity within the area of myometrial invasion. Imaging can also reveal extension into adjacent structures. Visualization of a pelvic mass on abdominal imaging is often the precipitant for further workup, and a definitive diagnosis is made on a hysterectomy specimen [3]. Through histopathologic examination of hysterectomy specimens, immunohistochemical markers such as CD10, vimentin, estrogen receptor (ER), and progesterone receptor (PR) can aid in confirming the diagnosis [4].

In this patient's case, a pelvic mass was first discovered on abdominal imaging performed to determine the etiology of her eosinophilia.

ESS is subclassified into low-grade (LGESS) and high-grade (high-grade endometrial stromal sarcoma [HGESS]) variants based on histological, immunophenotypic, and molecular criteria. HGESS, which was seen in this described case, is less common but exhibits more aggressive behavior, including a greater propensity for metastasis and recurrence, and is associated with poorer survival outcomes.

Peripheral eosinophilia is an atypical presentation in solid tumors and is rarely associated with uterine malignancies. While it has been documented in certain cancers like leiomyosarcoma and gastrointestinal tumors, there are no published reports directly linking eosinophilia to ESS [5, 6]. Eosinophils are white blood cells (WBCs) that can play both pro- and antitumor roles through cytokine release, immune modulation, and cytotoxic activity, though the mechanisms of their involvement in malignancies continue to be an area of ongoing investigation.

This case report describes a rare presentation of HGESS in a postmenopausal patient with unexplained peripheral eosinophilia, emphasizing the need to consider malignancy in the differential diagnosis of eosinophilia. It also explores the potential relationship between eosinophilia and tumor biology in uterine sarcomas, contributing to the limited existing literature on this novel association.

## 2. Case Presentation

A 66-year-old female with Type 2 diabetes mellitus, hypertension, depression, anxiety, and benzodiazepine dependence presented with generalized weakness, fatigue, and urinary incontinence. On presentation, laboratory findings were significant for a WBC count of 47.1 B/L, with 72% segmented neutrophils (absolute neutrophil count 35.18 B/L) and 16% eosinophils (absolute eosinophil count 8.93 B/L) and hemoglobin 11.9 g/dL. Her physical exam was insignificant. Imaging was only suspicious for a uterine mass seen on CT abdomen and pelvis with contrast during a previous hospital admission, measuring 7.8 cm. At the time, the mass was thought to represent a degenerating fibroid, but pelvic ultrasound was recommended for further evaluation as it had a centrally necrotic appearance. However, the patient was soon lost to follow-up.

Hematology, infectious disease, rheumatology, dermatology, and gynecology were consulted. Microbiological studies, including bacterial studies (*Rickettsia* IgG/IgM, *Treponema pallidum* antibody, and *Bartonella* antibody), viral studies (HIV), fungal studies (*Aspergillus* IgG/antigen, *Blastomyces* antibody, and *Coccidioides* antibody), parasitic studies (*Schistosoma* IgG, *Strongyloides* IgG, stool ova, and parasite), blood cultures, urine cultures, and transesophageal echocardiogram, were unrevealing for an infectious cause for eosinophilia. Antinuclear antibodies were negative, and complement levels were within normal limits. Urinalysis showed no significant findings.

**Figure 1:**
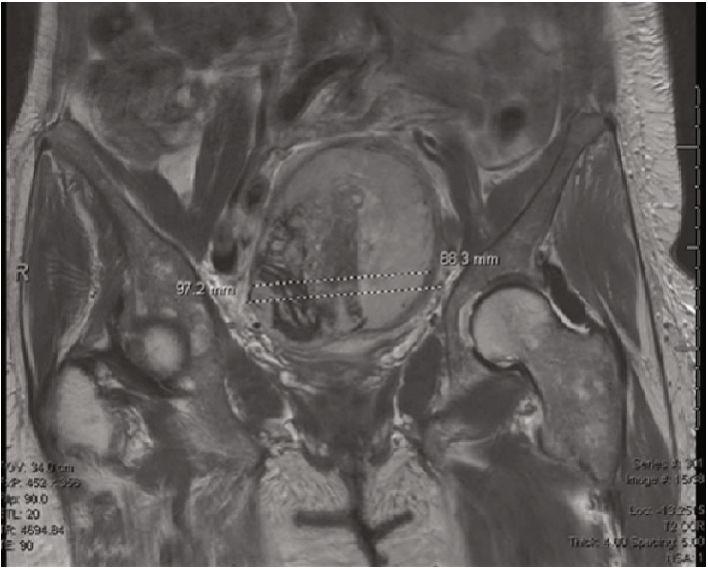
MRI pelvis depicting pelvic mass, later confirmed with pathology to be HGESS.

With the exhaustive workup remaining negative, the etiology of peripheral eosinophilia and leukocytosis was suspected to be due to the uterine mass previously seen on imaging. Repeat abdominal MRI characterized this as an enhancing mass within the myometrium with central necrosis. The mass had increased in size from the previous CT abdomen and pelvis performed 16 months prior, now measuring 9.8 × 8.2 × 8.8 cm, which was atypical in a postmenopausal woman and was concerning for malignancy (Figure 1). The patient underwent a total abdominal hysterectomy with bilateral salpingo-oophorectomy, partial upper vaginectomy, and lysis of adhesions. Intraoperatively, the uterine mass was found to be adherent to the anterior rectal wall, and colorectal surgery was consulted for assistance. Flexible sigmoidoscopy showed no intraluminal involvement. Low anterior resection with side-to-end stapled colorectal anastomosis was performed, and repeat flexible sigmoidoscopy with leak test was negative. A morcellator was not used. There was no visualization of residual tumor tissue.

**Figure 2:**
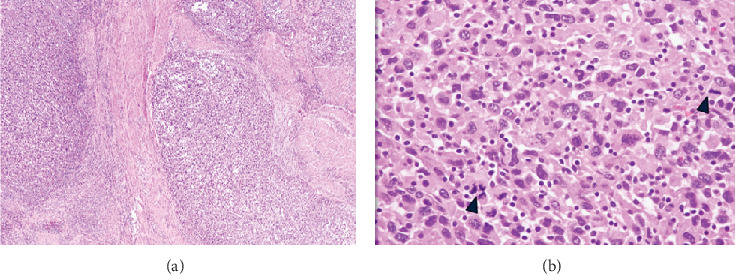
High-grade uterine sarcoma, H&E stain. (a) Low-power view of the infiltrative sarcoma with characteristic finger-like projections into the myometrium. (b) High-power view of the sarcoma with marked nuclear pleomorphism, prominent cytologic atypia, and increased mitotic figures (arrowhead).

After the surgery, her leukocytosis and eosinophilia rapidly resolved, with WBC decreasing from 57.1 B/L preoperatively to 26.8 B/L immediately after. Within 4 days after the hysterectomy, her WBC count had normalized to 10.4 B/L. While her CBC differential was not trended daily, there was a recorded resolution of the eosinophilia, from an absolute eosinophil count of 9.04 B/L (20.9% of WBC count) preprocedure to an absolute eosinophil count of 0.66 B/L (4.8% of the total WBC count) 20 days postprocedure (normal absolute eosinophil count: 0.00–0.70 B/L). The pathology of the uterus, cervix, fallopian tubes, ovaries, upper vagina, and sigmoid colon all showed high-grade uterine sarcoma with extensive lymphovascular invasion, FIGO Stage IIb. All 21 lymph nodes sampled were negative for tumor. As the pathology revealed a high-grade uterine sarcoma, this supported the diagnosis of HGESS (Figure 2). Adjuvant therapy was recommended but not pursued, as the patient was not amenable.

One month after the initial presentation, the patient returned with symptoms of nausea and vomiting and was found to have recurrent, rising eosinophilia with 19% eosinophils and 11.72 absolute eosinophils (WBC 61.7 B/L). A CT of the abdomen and pelvis revealed a 10 cm metastatic lesion located posterior to the stomach, along with new liver metastases causing bowel compression, indicative of Stage 4B disease. The patient elected to transition to inpatient hospice, as she did not want to pursue additional treatment.

## 3. Discussion

This case illustrates a rare presentation of HGESS in a patient with marked peripheral eosinophilia. While eosinophilia has been a documented atypical manifestation in other uterine malignancies such as leiomyosarcoma and other solid tumors, there are no other reported cases of peripheral eosinophilia as an atypical manifestation of ESS, specifically HGESS [7, 8]. This patient represents a unique case of HGESS that initially appeared clinically as peripheral eosinophilia with an unknown source. The imaging finding of a solid pelvic mass in the setting of marked peripheral eosinophilia raised suspicion for malignancy, which was confirmed with further evaluation of the tumor's pathology.

Immunohistochemistry can be used to confirm the diagnosis of ESS. HGESS tumor cells display cellular atypia, increased mitosis, infiltration, and necrosis with immunohistochemical results showing that most tumor cells are positive for CD10, vimentin, PR, and ER [4]. Our patient's pathology was typical of ESS but with no evidence of eosinophilic infiltration. Immediately after the removal of the uterine mass, her WBC count and eosinophilia down trended and resolved, which further strengthened the proposed association.

The mechanism responsible for eosinophilia is not fully understood. Secondary eosinophilia may be due to a paraneoplastic phenomenon, systemic effects of an immune response to disseminated malignancy, or a secondary reaction to extensive tumor necrosis [9]. Eosinophils have been described to exert both protumorigenic and antitumorigenic functions. The mechanisms by which they exert protumorigenic actions in some cancers have been attributed to the increased production of protumorigenic cytokines, remodeling of the extracellular matrix, and disruption of tumor vasculature [10]. This contrasts with literature that suggests eosinophils likely function as effector cells to increase host antitumor responses [11]. In the presence of cancer, activated eosinophils in tissues can undergo partial degranulation. The release of substances such as MBP1—which disrupts the integrity of lipid bilayers of cells—and EPX—which induces oxidative stress—can induce cell death, creating a profound antitumor effect within the tumor microenvironment.

As such, eosinophils can directly and indirectly affect tumor biology through their ability to express immune regulatory cytokines and thereby influence innate and adaptive immunity. However, further research is needed to elucidate both the positive and negative effects that eosinophils create on tumor biology, especially regarding endometrial sarcomas. While it is unable to be conclusively determined whether the eosinophilia described in this case was secondary to an antitumor or protumor mechanism, the cumulative evidence provided by similar case reports on uterine malignancies further suggests an association between marked eosinophilia and solid tumors [5, 12, 13].

The prognosis of ESS varies depending on the grade of the malignancy. ESS may be subclassified into low grade (LGESS) and high grade (HGESS) depending on morphologic, immunophenotypic, and molecular features. HGESS, seen in this patient's case, is rarer than LGESS and often has rapid development. It is more prone to recurrence and metastatic spread, particularly to the lymph nodes, bones, and lungs as seen in this case. LGESS typically portends an excellent prognosis, whereas HGESS has poorer outcomes. In one observational retrospective cohort study, HGESS was associated with a median survival of 19.9 months, with a 5-year survival of 32.6% compared to 90.5% seen in LGESS, highlighting the disparate prognoses [14]. Negative prognostic factors include increased age, tumor size, distant or nodal metastasis, omission of lymphadenectomy, and pathologically positive surgical margins. The most common treatment is total hysterectomy and bilateral salpingo-oophorectomy, whereas hormone therapy is often recommended for LGESS [15]. Adjuvant chemotherapy and radiotherapy have been associated with increased survival in HGESS [14].

Unfortunately, the described case in this report demonstrated the poor prognosis associated with HGESS. This patient re-presented 1 month later with nausea and vomiting and was found to have recurrent up trending eosinophilia. CTAP revealed metastatic lesions posterior to the body of the stomach, measuring 10 cm. She was also found to have new metastases in the liver as well, causing bowel compression. This finding further supports the association of tumor-associated blood eosinophilia with tumor spread and poor prognosis [5]. Ultimately, the patient elected to pursue inpatient hospice care.

In conclusion, this case report underscores the importance of considering eosinophilia as a potential indicator of underlying malignancy, particularly when a pelvic mass is present, and highlights the complexities of diagnosing and managing atypical presentations of uncommon malignancies. The strengths of this case report include highlighting an unusual presentation of a noncommon malignancy, which may inform future workup strategies in similar clinical scenarios. The case broadens the differential diagnosis of unexplained eosinophilia and highlights the importance of considering occult malignancy in persistent cases. While this appears to be the first reported case of ESS presenting with peripheral eosinophilia, the absence of similar reports limits broader generalizability. Further cases are needed to establish any potential causal or pathophysiologic link.

Recognizing and investigating the rare association between marked peripheral eosinophilia and ESS, particularly HGESS, will allow for further understanding of the disease process and its systemic effects. Furthermore, identifying atypical hematologic findings as manifestations of malignancy can facilitate early diagnosis and treatment. Our case highlights eosinophilia as an associated finding of HGESS, a novel finding in the literature. This also underscores the need for further research to strengthen this association and unveil the molecular underpinnings between marked peripheral eosinophilia and uterine malignancy.

## Data Availability

Data sharing is not applicable to this article as no new data were created or analyzed in this study.
